# Prognostic factors and overall survival in a 15-year followup of patients with malignant salivary gland tumors: a retrospective analysis of 193 patients

**DOI:** 10.1016/j.bjorl.2020.06.016

**Published:** 2020-08-04

**Authors:** Osias Vieira de Oliveira Filho, Talita Jordânia Rocha do Rêgo, Felipe Herbert de Oliveira Mendes, Thinali Sousa Dantas, Maria do Perpétuo Socorro Saldanha Cunha, Cássia Emanuella Nóbrega Malta, Paulo Goberlânio de Barros Silva, Fabrício Bitu Sousa

**Affiliations:** a Universidade Federal do Ceará, Faculdade de Farmácia, Odontologia e Enfermagem, Divisão de Patologia Oral, Fortaleza, CE, Brazil; b Centro Universitário Christus (Unichristus), Departamento de Odontologia, Fortaleza, CE, Brazil; c Hospital Haroldo Juaçaba, Escola de Oncologia do Ceará, Fortaleza, CE, Brazil

**Keywords:** Survival analysis, Oral neoplasms, Parotid neoplasms, Submandibular gland neoplasms, Sublingual gland neoplasms

## Abstract

**Introduction:**

Malignant tumors of the salivary glands are uncommon pathological entities, representing less than 5% of head and neck neoplasms. The prognosis of patients with malignant tumors of the salivary glands is highly variable and certain clinical factors can significantly influence overall survival.

**Objective:**

To analyze the clinicopathologic and sociodemographic characteristics that influence survival in patients with malignant tumors of the salivary glands

**Methods:**

This retrospective study analyzed sex, age, race, education level, tumor location, tumor size, lymph node involvement, distant metastasis, margin status, treatment type, marital status, method of health care access and 15-year overall survival in 193 patients with malignant tumors of the salivary glands. The X², log-rank Mantel-Cox, multinomial regression and Cox logistic regression tests were used (SPSS 20.0,*p* < 0.05).

**Results:**

The most common histological types were adenocarcinoma (32.1%), adenoid cystic carcinoma (31.1%) and mucoepidermoid carcinoma (18.7%). The 15-year overall survival rate was 67.4%, with a mean of 116 ± 6 months. The univariate analysis revealed that male sex (*p* = 0.026), age > 50 years (*p* = 0.001), referral origin from the public health system (*p* = 0.011), T stage (*p* =  0.007), M stage (*p* <  0.001), clinical stage (*p* <  0.001), compromised surgical margins (*p* =  0.013), and chemotherapy (*p* <  0.001) were associated with a poor prognosis. Multivariate analyses also showed that age > 50 years was independently associated with a poor prognosis (*p* =  0.016). The level of education was the only factor more prevalent in older patients (*p* =  0.011).

**Conclusion:**

Patients with malignant tumors of the salivary glands older than 50 years have a worse prognosis and an independent association with a low education level.

## Introduction

Malignant tumors of the salivary glands (MTSG) are uncommon pathological entities, representing less than 5% of head and neck neoplasms.^1^ The prognosis of patients with MTSG varies greatly, and some clinical factors significantly influence disease-free time and overall survival. The survival rate of these tumors ranges from 19.6% to 84.7%, depending on the histological type and anatomical site and particularly the therapy of choice.^2^, ^3^, ^4^, ^5^

Epidemiologically, these neoplasms are more frequent in the parotid gland in middle-aged female adults and are represented primarily by mucoepidermoid carcinoma (MEC) and adenoid cystic carcinoma (ACC).^6^ Despite their high prevalence in the parotid gland, these lesions are heterogeneously distributed considering their topographic distribution. Only 25% of parotid gland tumors are malignant; however, submandibular gland tumors and minor salivary gland tumors represent 50% and 81% of malignancies, respectively.^7^

Survival studies have reported different frequencies and distributions of sociodemographic factors such as age, sex and race.^8^, ^9^, ^10^ These variables become difficult to compare among studies because they are highly diverse and undergo intense reclassifications.^11^ Additionally, the prognosis is directly influenced by the site of involvement, the staging of the tumor and its histological type. The behavior of salivary gland tumors still has little predictability due to the lack of well-delineated prognostic factors, which makes most of these tumors extremely aggressive, resulting in a poor prognosis.^2^, ^7^

Numerous factors seem to be associated with the poor prognosis of MTSG, and this is very important to consider during diagnosis to guide the appropriate treatment. Indeed, different populations have individual and, sometimes, very specific risk factors in the development of this set of lesions.^12^, ^13^ Similarly, the therapeutic approach of the treatment center may also influence the prognosis of patients with these lesions.^4^

Nevertheless, the rarity of these tumors makes it challenging to conduct case-control, cohort, and clinical trials. This makes cross-sectional observational studies an important source of information about this type of lesion.^6^, ^9^, ^12^, ^14^ Thus, the objective of this study was to characterize, based on a retrospective analysis, malignant tumors of the salivary glands and to identify clinical and sociodemographic factors that influence the survival of this group of patients.

## Methods

### Study design and ethical precepts

This was an observational, retrospective, cross-sectional study in which clinical and pathological data were collected from patients with malignant tumors of the salivary glands diagnosed at Haroldo Juaçaba Hospital - Ceará Cancer Institute over 15 years (January 1, 2000 to December 31, 2014). This study was submitted to the Research Ethics Committee (CEP) of the Haroldo Juaçaba Hospital - Ceará Cancer Institute and was accepted under opinion number 1,203,732.

### Data sampling and survey

We evaluated the biopsy reports and medical records of patients whose entry into the service was between January 1, 2000 and December 31, 2014. The classification was based on guidelines from the World Health Organization in 2017.^11^ Data on histological type, sex, age, race/ethnicity, patient origin (rural area/metropolitan area), patient education level, hospital access mode (public or private service), primary tumor location (large or small salivary glands; if small, which region of the oral cavity), Tumor size (T), the presence of lymph Node metastasis (N), the presence of distant Metastasis (M), staging, and type of treatment (surgery, radiotherapy, chemotherapy and their associations) were analyzed.

The overall survival status was also assessed, and the survival time (in months) was calculated by subtracting the date at the initial diagnosis from the date of last return to the hospital or death.^15^

### Statistical analysis

Descriptive data and death frequencies are expressed as absolute and percentage frequencies and were compared by Fisher's exact test or Pearson's Chi-Square test. Survival time was based on Kaplan-Meier curves and compared using the log-rank Mantel-Cox test (expressed as the mean and standard error).

We then used the forward stepwise model in which variables with *p* < 0.200 were added to the multivariate model. Survival curves introduced in the model were analyzed using Cox regression, and subsequent categorical data were analyzed using multinomial logistic regression. All analyses were performed using Statistical Packing for Social Sciences (SPSS) software 17.0 (SPSS, Chicago, IL, USA) (*p* < 0.05).

## Results

### Sample characterization: histological types

From 2000 to 2014, a total of 193 patients with malignant tumors of the salivary glands were diagnosed at Haroldo Juaçaba Hospital - Ceará Cancer Institute. Of these, there were 62 (32.1%) diagnoses of adenocarcinoma, NOS (AC-NOS), followed by 60 (31.1%) diagnoses of ACC, 36 (18.7%) diagnoses of MEC and 35 (18.1%) diagnoses of another tumor: 11 acinic cell carcinomas, 9 epithelial-myoepithelial carcinoma, 5 salivary duct carcinomas, 4 intraductal carcinomas, 3 myoepithelial carcinomas, 2 lymphoepithelial carcinomas and 1 carcinosarcoma.

### Sample characterization: sociodemographic factors and overall survival

**Figure 1:**
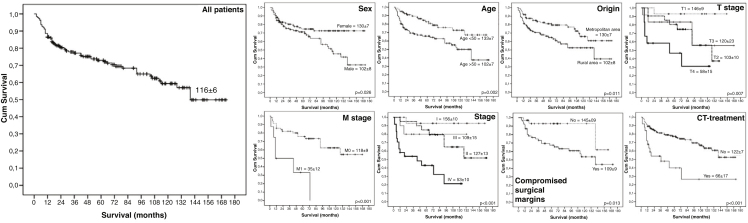
Fifteen-year survival curves (2000−2014) of patients diagnosed with malignant salivary gland neoplasms at Haroldo Juaçaba Hospital - Ceará Cancer Institute (log-rank Mantel-Cox test).

Over the 15 years of evaluation, the overall survival rate was 67.4% (n = 130), with a mean survival time of 116 ± 6 months. Among the patients surveyed, 99 (55.1%) were female, and 94 (48.7%) were male. In male patients, the total number of patients alive (*p* =  0.011) and the mean survival time (*p* = 0.026) were significantly lower than those in female patients. Most patients were older than 50 years (n = 112, 58.0%), and patients older than this cut-off point had a higher rate of death (*p* =  0.003) and shorter survival times (*p* =  0.001) (Table 1, Fig. 1).

**Table 1:** Influence of sociodemographic profile on the death frequency and survival time of patients with malignant lesions of salivary glands diagnosed and treated at Haroldo Juaçaba Hospital - Ceará Cancer Institute between 2000 and 2014.

		Death			Average survival	
	Total	Alive	Death	*p*-value		*p*-value
**Sex**						
Male	94 (48.7%)	55 (42.3%)	39 (61.9%)^a^	0.011^a^	102 ± 8	0.026^a^
Female	99 (51.3%)	75 (57.7%)^a^	24 (38.1%)		130 ± 7^b^	
**Age**						
Up to 50 years	81 (42.0%)	64 (49.2%)^a^	17 (27.0%)	0.003^a^	133 ± 7^b^	0.001^a^
Over 50 years	112 (58.0%)	66 (50.8%)	46 (73.0%)^a^		102 ± 7	
**Race**						
White	75 (40.8%)	52 (43.0%)	23 (36.5%)	0.491	110 ± 7	0.442
Dark skin	109 (59.2%)	69 (57.0%)	40 (63.5%)		116 ± 8	
**Education level**						
Illiterate / Incomplete elementary school	78 (56.9%)	43 (49.4%)	35 (70.0%)^a^	0.019^a^	110 ± 7	0.212
Complete Elementary school or higher	59 (43.1%)	44 (50.6%)^a^	15 (30.0%)		116 ± 8	
**Origin**						
Metropolitan area	97 (50.3%)	73 (56.2%)^a^	24 (38.1%)	0.019^a^	130 ± 7^b^	0.011^a^
Rural area	96 (49.7%)	57 (43.8%)	39 (61.9%)^a^		102 ± 8	
**Marital status**						
Married	52 (27.1%)	39 (30.2%)	13 (20.6%)	0.358	125 ± 10	0.594
Unmarried	111 (57.8%)	72 (55.8%)	39 (61.9%)		114 ± 7	
Others	29 (15.1%)	18 (14.0%)	11 (17.5%)		102 ± 12	
**Referral**						
Public Health System	96 (77.4%)	59 (73.8%)	37 (84.1%)	0.188	109 ± 10	0.278
Private Health System	28 (22.6%)	21 (26.3%)	7 (15.9%)		106 ± 7	
**Family history**						
Yes	28 (46.7%)	22 (50.0%)	6 (37.5%)	0.391	91 ± 8	0.747
No	32 (53.3%)	22 (50.0%)	10 (62.5%)		108 ± 9	
**Alcoholism**						
Yes	52 (88.1%)	38 (88.4%)	14 (87.5%)	1.000	105 ± 8	1.000
No	7 (11.9%)	5 (11.6%)	2 (12.5%)		37 ± 6	
**Smoking**						
Yes	43 (78.2%)	35 (83.3%)	8 (61.5%)	0.129	102 ± 6	0.070
No	12 (21.8%)	7 (16.7%)	5 (38.5%)		73 ± 13	

a *p* < 0.05, Chi-Square or Fisher's exact test; data are expressed as the absolute frequency and percentage.

b *p* <  0.05, Log-rank Mantel-Cox test; data are expressed as the mean and standard error devised by Kaplan-Meier curves.

Most of the cohort was of mixed ethnicity (n = 109, 59.2%). Patients with a low education level (n = 78, 56.9%) also accounted for the majority. Despite having a higher mortality rate (*p* =  0.019), these patients had the same survival time as patients with a higher level of education (*p* = 0.212) (Table 1, Fig. 1).

Patients living in a metropolitan area (n = 97, 50.3%) showed lower mortality rates and longer survival times compared to those living in a rural area (n = 96, 49.7%). Living in a rural area also had a significant influence on mortality (*p* =  0.019) and survival time (*p* =  0.011) (Table 1, Fig. 1).

Other sociodemographic factors studied were marital status, with a higher prevalence of unmarried people (n = 111, 57.8%), and hospital access mode, with a higher prevalence from the public service (n = 96, 77.4%). Cancer family history was absent in most patients (n = 32, 53.3%), and previous use of alcohol and smoking were present in most patients (n = 52, 88.1% and n = 43, 78.2%, respectively). However, these factors did not influence mortality or survival time (Table 1, Fig. 1).

### Clinicopathological factors and overall survival

Most tumors were from major salivary glands (n = 127, 65.8%), but this factor did not influence patient survival time (*p* = 0.073 and *p* = 0.097). The most common histological type, AC-NOS, had the highest mortality rate (n = 27, 42.9%), followed by ACC (n = 21, 33.3%) and other tumors (n = 10, 15.9%). The lowest mortality rate was observed in patients with MEC (n = 5, 7.9%) (*p* = 0.023). There were no differences on overall survival between these major histologic tumors types (*p* = 0.054) (Table 2).

**Table 2:** Influence of the clinical-pathological and therapeutic profile on the mortality rate and survival time of patients with malignant lesions of salivary glands diagnosed and treated at Haroldo Juaçaba Hospital - Ceará Cancer Institute between 2000 and 2014.

		Death			Average Survival	
	Total	Alive	Death	*p*-value		Total
**Location**						
Minor	66 (34.2%)	50 (38.5%)	16 (25.4%)	0.073	130 ± 9	0.097
Major	127 (65.8%)	80 (61.5%)	47 (74.6%)		110 ± 7	
**Histological type**						
AC-NOS	61 (32.1%)	35 (26.9%)	27 (42.9%)^a^	0.023^a^	101 ± 10	0.054
ACC	60 (31.1%)	39 (30.0%)	21 (33.3%)		114 ± 9	
MEC	36 (18.7%)	31 (23.8%)^a^	5 (7.9%)		130 ± 8^b^	
Others	36 (18.7%)	25 (19.2%)	10 (15.9%)		109 ± 11	
**T**						
1	14 (23.7%)	13 (32.5%)^a^	1 (5.3%)	0.038^a^	146 ± 9^b^	0.007^a^
2	21 (35.6%)	13 (32.5%)	8 (42.1%)		103 ± 10	
3	12 (20.3%)	9 (22.5%)	3 (15.8%)		120 ± 23	
4	12 (20.3%)	5 (12.5%)	7 (36.8%)^a^		58 ± 15	
**N**						
N0	45 (81.8%)	32 (84.2%)	13 (76.5%)	0.479	124 ± 10	0.342
N+	9 (16.4%)	6 (15.8%)	4 (23.5%)		81 ± 16	
**M**						
0	56 (87.5%)	38 (95.0%)^a^	18 (75.0%)	0.044^a^	118 ± 9^b^	<0.001
1	8 (12.5%)	2 (5.0%)	6 (25.0%)^a^		35 ± 12	
**Stage**						
1	16 (22.9%)	15 (33.3%)^a^	1 (4.0%)	0.002^a^	156 ± 10^b^	<0.001
2	20 (28.6%)	13 (28.9%)	7 (28.0%)		127 ± 13	
3	10 (14.3%)	8 (17.8%)	2 (8.0%)		109 ± 15	
4	24 (34.3%)	9 (20.0%)	15 (60.0%)^a^		53 ± 10	
**Surgery**						
No	100 (51.8%)	65 (50.0%)	35 (55.6%)	0.469	111 ± 8	0.325
Yes	93 (48.2%)	65 (50.0%)	28 (44.4%)		121 ± 8	
**Compromised surgical margins**						
No	32 (34.4%)	29 (44.6%)^a^	3 (10.7%)	0.002	145 ± 9	0.013
Yes	61 (65.6%)	36 (55.4%)	25 (89.3%)^a^		109 ± 9	
**RT**						
No	71 (36.8%)	50 (38.5%)	21 (33.3%)	0.488	122 ± 9	0.598
Yes	122 (63.2%)	80 (61.5%)	42 (66.7%)		113 ± 7	
**CT**						
No	168 (87.0%)	119 (91.5%)^a^	49 (77.8%)	0.008^a^	122 ± 7^b^	<0.001
Yes	25 (13.0%)	11 (8.5%)	14 (22.2%)^a^		66 ± 17	

RT, Radiotherapy; CT, Chemotherapy.

a *p* < 0.05, Chi-Square or Fisher's exact test; data are expressed as the absolute frequency and percentage.

b *p* < 0.05, Log-rank Mantel-Cox test; data are expressed as the mean and standard error devised by Kaplan-Meier curves.

The patients were in homogeneous stages: T1 (n = 14, 23.7%), T2 (n = 21, 35.6%), T3 and T4 (both with n = 12, 20.3%). Patients in T4 stage showed the highest mortality rate (*p* =  0.038) and a short survival time (*p* =  0.007). Most patients had no lymph node metastasis (n = 45, 81.8%), and this factor did not significantly influence mortality (*p* =  0.479) or survival time (*p* =  0.342). We observed the presence of metastases only in 8 patients (12.5%). This factor significantly increased the mortality rate (*p*  = 0.002) and reduced survival time (*p* <  0.001).

The patients of rural areas had a slight increase in prevalence of T3/4 tumors (n = 18, 52.9% vs. n = 14, 40.0%; *p* = 0.281) and M1 tumors (n = 7, 21.2% vs. n = 4, 11.1%; *p* =  0.330), however these variables were not significantly influenced by the regional origin of the patient.

Of the treatments performed, radiotherapy was the most commonly used (n = 122, 63.2%), followed by surgery (n = 93, 48.2%) and chemotherapy (n = 25, 13.0%). The patients with compromised surgical margins and patients who underwent chemotherapy had a high mortality rate (*p* =  0.002 and *p* = 0.008, respectively) and short survival time (*p* =  0.013 and *p* <  0.001, respectively) (Table 2, Fig. 1).

### Survival analysis: age and education as factors directly and indirectly associated

In the multivariate survival analysis, only patient age over 50 years significantly and independently influenced survival (*p* =  0.016). Patients over 50 years of age had a risk of death 9.967 times higher than patients younger than this cut-off (95% CI 5.772−171.507) (Table 3).

**Table 3:** Multivariate survival analysis of patients with malignant salivary gland lesions diagnosed and treated at Haroldo Juaçaba Hospital - Ceará Cancer Institute between 2000 and 2014.

	*p*-value	Adjusted HR	95% CI	
Sex	0.833	1.230	0.178	8.458
Age	0.016^a^	9.967	5.772	171.507
Origin	0.062	6.217	0.628	26.731
Smoking	1.000	0.470	0.008	7.421
Location	0.052	4.021	0.939	17.287
Histological type	0.053	0.193	0.036	1.024
T	0.082	5.471	0.987	20.512
M	0.087	0.397	0.016	7.779
Staging	0.080	0.021	0.001	1.838
Compromised surgical margins	0.999	1.390	0.123	13.912
CT	0.089	1.454	0.117	1.808

HR, Hazard Risk; CI, Confidence Interval (95%); CT, Chemotherapy.

a *p* < 0.05, Cox regression forward stepwise model.

Patients over 50 years old had a higher prevalence of males (*p* =  0.030), those with a low education level (*p*  = 0.002), unmarried individuals (*p* <  0.001), and nonsmokers (*p* =  0.002) and were more often treated with radiotherapy (*p* =  0.029) and chemotherapy (*p* =  0.018) (Table 4). The factor that was independently associated with age was the level of education (*p* =  0.011), which was 0.047 times lower in patients over 50 years old (95% CI 0.004−0.503) (Table 5).

**Table 4:** Influence of age on sociodemographic, clinical-pathological, and therapeutic profiles of patients with malignant lesions of salivary glands diagnosed and treated at Haroldo Juaçaba Hospital - Ceará Cancer Institute between 2000 and 2014.

	Age		*p*-value
	Up to 50 years	Over 50 years	
**Sex**			
Male	32 (39.5%)	62 (55.4%)^a^	0.030
Female	49 (60.5%)^a^	50 (44.6%)	
**Race**			
White	33 (43.4%)	42 (38.9%)	0.538
Dark skin	43 (56.6%)	66 (61.1%)	
**Education level**			
Illiterate / Incomplete elementary school	20 (39.2%)	58 (67.4%)^b^	0.002^a^
> Elementary school	31(60.8%)^a^	28 (32.6%)	
**Origin**			
Metropolitan area	44 (54.3%)	53 (47.3%)	0.337
Rural area	37 (45.7%)	59 (52.7%)	
**Marital status**			
Married	35 (43.2%)^a^	17 (15.3%)	<0.001
Singles	41 (50.6%)	70 (63.1%)	
Divorced / Separate	5 (6.2%)	24 (21.6%)^a^	
**Referral**			
Public Health System	43 (79.6%)	53 (75.7%)	0.605
Private Health System	11 (20.4%)	17 (24.3%)	
**Family history**			
Yes	9 (47.4%)	19 (46.3%)	0.941
No	10 (52.6%)	22 (53.7%)	
**Alcoholism**			
Yes	22 (95.7%)	30 (83.3%)	0.229
No	1 (4.3%)	6 (16.7%)	
**Smoking**			
Yes	21 (100.0%)^a^	22 (64.7%)	0.002^a^
No	0 (0.0%)	12 (35.3%)^a^	
**Location**			
Minor	28 (34.6%)	38 (33.9%)	1.000
Major	53 (65.4%)	74 (66.1%)	
**Histological type**			
NOS-AC	19 (23.5%)	43 (38.4%)	0.086
ACC	28 (34.6%)	32 (28.6%)	
MEC	20 (24.7%)	16 (14.3%)	
Others	14 (17.3%)	21 (18.8%)	
**T**			
1	10 (35.7%)	4 (12.9%)	0.210
2	9 (32.1%)	12 (38.7%)	
3	4 (14.3%)	8 (25.8%)	
4	5 (17.9%)	7 (22.6%)	
**N**			
N0	24 (85.7%)	21 (77.8%)	0.503
N+	4 (14.3%)	6 (22.2%)	
**M**			
0	26 (86.7%)	30 (88.2%)	1.000
1	4 (13.3%)	4 (11.8%)	
**Staging**			
1	11 (33.3%)	5 (13.5%)	0.164
2	10 (30.3%)	10 (27.0%)	
3	4 (12.1%)	6 (16.2%)	
4	8 (24.2%)	16 (43.2%)	
**Surgery**			
No	41 (50.6%)	59 (52.7%)	0.884
Yes	40 (49.4%)	53 (47.3%)	
**Compromised surgical margins**			
No	23 (57.5%)	38 (71.7%)	0.201
Yes	17 (42.5%)	15 (28.3%)	
**RT**			
No	37 (45.7%)^a^	34 (30.4%)	0.029^a^
Yes	44 (54.3%)	78 (69.6%)^a^	
**CT**			
No	76 (93.8%)^a^	92 (82.1%)	0.018^a^
Yes	5 (6.2%)	20 (17.9%)^a^	

RT, radiotherapy; CT, chemotherapy.

a *p* < 0.05, Chi-Square or Fisher's exact test; data are expressed as the absolute frequency and percentage.

**Table 5:** Multivariate analysis of the influence of age on sociodemographic, clinical-pathological, and therapeutic characteristics of patients with malignant lesions of salivary glands diagnosed and treated at Haroldo Juaçaba Hospital - Ceará Cancer Institute between 2000 and 2014.

	*p*-value	Adjusted OR	95% CI	
Sex	0.414	0.454	0.068	3.021
Education level	0.011^a^	0.047	0.004	0.503
Marital status	0.134	0.143	0.011	1.826
Smoking	0.998	0.123	0.012	3.213
Histologic type	0.201	0.119	0.005	3.106
Staging	0.315	3.058	0.345	27.109
RT	0.715	0.689	0.094	5.074
CT	0.212	7.793	0.311	195.466

OR, Odds Ratio; CI, Confidence Interval (95%); RT, Radiotherapy; CT, Chemotherapy.

a *p* < 0.05, multinomial logistic regression forward stepwise model.

## Discussion

MTSG are a heterogeneous group of rare tumors in which the predictors of mortality are uncertain, and death can occur up to decades after initial treatment, which proves the tendency for late recurrence.^8^, ^14^ Therefore, to obtain reliable information on prognostic factors, it is necessary to follow up patients for many years. This is probably the reason why the vast majority of studies are retrospective.^6^, ^9^, ^12^, ^14^

The present study observed an overall survival rate of 67.4% and a survival time of 116 ± 6 months in a 15-year follow-up. Other authors have reported lower 15-year overall survival rates, ranging from 39.8% to 42%.^12^, ^13^ Even in 10 and 5 year estimates, lower rates have been reported, ranging from 19.6% to 51% and 35.3% and 65.9%, respectively.^5^, ^8^, ^9^, ^12^^,^^13^, ^16^ Higher values have also been presented in the literature.^4^, ^17^ Ouyang et al.^4^ presented 5, 10, and 15-year overall survival rates of 84.7%, 70.8%, and 34%, respectively. Monteiro et al.^17^ presented a slightly higher value than the one we found (71% survival after 5 years). These differences may be related to the histological variability of the samples and the therapeutic approach employed at each center.^9^, ^17^

MTSG proved to be a heterogeneous group, and the histological variability of the sample was compatible with previously reported data.^3^, ^5^, ^10^, ^12^^,^^13^, ^17^, ^18^ In the present study, AC was the most common histological type, followed by ACC, MEC and nine other different tumors.

Our data revealed no different prognoses for histological types. However, Baddour et al.^19^ and Kokemueller et al.^13^ demonstrated higher survival rates at 5, 10, and 15 years for MEC in relation to AC.

In our study, there was no sex predominance, which is in agreement with other findings.^13^, ^17^, ^20^, ^21^, ^22^ However, in previous Brazilian studies, a predilection for females^6^, ^23^, ^24^, ^25^ was observed when analyzing a variety of tumors. A large Brazilian study, with 2292 cases, demonstrated a female:male ratio of 1.5:1; but when only ACC was described, there was a slight predominance in males (M:F = 1.22:1).^26^ Predilection for the male sex was also demonstrated when evaluating malignant and benign tumors together, but these findings are supported by a smaller number of evaluated cases.^27^

The univariate analysis showed a higher death frequency in males. This finding is in agreement with several other previous studies.^5^, ^8^, ^12^, ^16^^,^^21^, ^28^ Iwata et al.^16^ found, in a univariate analysis, that the female sex is a protective factor, bringing a 50% increase in the 5-year survival rate and a 40% increase in the 10-year survival rate. This fact remained in the multivariate analysis, in which the female sex brought a 47% increase in the 5-year survival rate and a 40% increase in the 10-year survival rate.^16^ Interestingly, Baddour et al.^19^ found that sex is not an independent prognostic factor that influences the overall survival of these patients, similar to our study.

We found an influence of the origin of referral of the patients on overall survival. Patients from rural locations showed a worse prognosis than patients from metropolitan regions. These findings resemble those from US studies.^16^, ^28^ Access to highly complex oncology services is an important prognostic factor;^28^ evidence confirmed by Iwata et al.^16^ showed that with an increase in population density, there was an improvement in survival at 5 and 10 years.

Advanced clinical stage, according to the AJCC's TNM classification, has been reported as a significant predictor of prognosis in patients with MTSG,^3^, ^12^, ^13^, ^16^, ^17^, ^18^, ^21^ which was confirmed in our study. Both the T and M stages showed a significant influence on the death frequency and patient survival, in agreement with the literature.^3^, ^16^, ^17^, ^18^, ^21^, ^26^ Moreover, stage IV showed the worst prognosis.

In addition, for therapy and prognosis, it is important to determine the status of surgical margins.^13^ Our data presented a decreased survival in patients with compromised surgical margins, similar to the current literature.^4^, ^12^, ^13^ Ouyang et al.^4^ also demonstrated margin involvement as an independent risk factor for distant metastasis. According to Lewis et al.,^29^ factors predictive of a partial response to definitive radiation include size greater than 4 cm, T4 stage cancer, and stage IV disease. Radiotherapy is mainly reserved for inoperable cases and for patients who refuse surgery.^13^, ^29^, ^30^ Most patients in our cohort did not undergo surgery, which could be related to the considerable number of T4 and stage IV cancers.

Moreover, in our study only chemotherapy was a significant predictor (patients treated with chemotherapy experienced shorter survival), similar to previous reports.^8^, ^13^ In fact, MTSG show a limited response to conventional chemotherapy, being usually reserved for palliative treatment.^13^, ^14^, ^30^

In the multivariate analysis, only age over 50 years independently influenced the survival of these patients. Several studies have confirmed the influence of age on the prognosis of malignant tumors in salivary glands.^4^, ^5^, ^8^, ^9^^,^^12^, ^16^, ^18^, ^19^^,^^28^ However, opposite results have also been found.^17^ Consistent with our findings, Iwata et al.^16^ showed that for each year, there is a 5% increase in the risk of death in 5-years (HR = 1.50, 95% CI 1.08 − 2.08) and 6% in the risk of death in 10-years (95% CI 1.04 − 1.08).

This finding may be partially explained by the fact that there is a tendency towards more aggressive and, in turn, more effective treatments in younger patients and because of the effect of age on mortality in general.^12^ In addition, older patients tend to have more comorbidities, which limits the treatment response and compromises tolerance to more aggressive treatments.^4^ In the cohort of Cheung et al.^8^ several comorbidities affected the survival of the patients evaluated, and Iwata et al.^16^ demonstrated through the Charlson Comorbidity Index^31^ that for every 1 point increase in the index, there is a 19% increase in the risk of death in the 5-year analysis and an 18% increase in the 10 year analysis.

In the distribution of patients regarding age, in the multivariate analysis, only the level of education showed an independent association with the prognosis of the patients evaluated, acting as a factor indirectly associated with a worse prognosis. This finding is in agreement with recent studies conducted to analyze the influence of socioeconomic variables on MTSG^16^, ^28^ In the study by Olarte et al.^28^ educational level influenced the survival of patients in the univariate analysis. In the study by Iwata et al.^16^ the multivariate analysis revealed an influence of the educational level on the increased risk of death at 5 and 10 years.

The results of the present study highlight the importance of age and education in MTSG prognosis. However, the study has some limitations. As a retrospective and unicentric study, it does not offer the highest level of clinical evidence. A significant bias related to high education level of the population treated in our center was observed. More educated patients are more likely to seek medical care earlier, thereby improving prognosis. Therefore, the application and generalization of our results should be viewed with caution. Additionally, it is necessary to consider the heterogeneity of the treatments applied over this long period and the loss of follow-up data. Nonetheless, this study evaluated 15-year follow-up data, which is a long observational time, and had a large sample of MTSG, strongly contributing to the recognition of prognostic factors in this rare group of tumors.

## Conclusion

Patients older than 50 years of age have a poor prognosis, with education being the main variable associated with this risk factor. Extensive multicenter studies and systematic reviews with meta-analyses are needed to better understand the prognosis of these malignancies to optimize treatment strategies and develop targeted therapies.

## Conflicts of interest

The authors declare no conflicts of interest.
